# Hyperbranched Poly(ether-siloxane)s Containing Ammonium Groups: Synthesis, Characterization and Catalytic Activity

**DOI:** 10.3390/polym12040856

**Published:** 2020-04-07

**Authors:** Paweł G. Parzuchowski, Aleksandra Świderska, Marlena Roguszewska, Karolina Rolińska, Dominik Wołosz, Mariusz Mamiński

**Affiliations:** 1 Faculty of Chemistry, Warsaw University of Technology, Noakowskiego 3, 00-664 Warsaw, Poland; aswiderska@ch.pw.edu.pl (A.Ś.); mroguszewska@ch.pw.edu.pl (M.R.); krolinska@ch.pw.edu.pl (K.R.); dominik.wolosz@onet.pl (D.W.); 2 Faculty of Chemistry, University of Warsaw, ul. Pasteura 1, 02-093 Warsaw, Poland; 3 Institute of Wood Sciences and Furniture, Warsaw University of Life Sciences – SGGW, Nowoursynowska 159, 02-787 Warsaw, Poland; mariusz_maminski@sggw.pl

**Keywords:** hyperbranched polymer, carbon dioxide, cycloaddition, polysiloxane, polyglycidol, catalysis, oxirane, ethylene carbonate

## Abstract

In this article we report an easy synthetic route towards hyperbranched polyglycerols (Amm-HBPGs) containing trimethylammonium groups and siloxane or hydroxyl end-groups. Siloxane derivatives of Amm-HBPGs were synthesized in an efficient five-step procedure including an anionic ring opening copolymerization of the phthalimide-epoxy monomer with glycidol, followed by reactions with allyl bromide, hydrosililation with hydrogenheptamethyltrisiloxane, hydrazinolysis of phthalimide groups and quaternization of resulting amine groups with methyl iodide. Hydroxyl derivatives were obtained by quaternization of previously reported aminated HBPG’s with methyl iodide. Polymeric products were characterized using various NMR techniques, FTIR, and elemental analysis. Both Amm-HBPGs were shown to be effective in catalysis of addition of CO_2_ to oxirane. The hydrophilic catalysts showed higher efficiency but synthesis of ethylene carbonate was accompanied by formation of small amounts of ethylene glycol. The siloxane-containing catalyst was easily separable from reaction mixture showing high potential in the process of converting carbon dioxide into valuable chemical raw materials.

## 1. Introduction

The CO_2_ concentration in the air is at its highest level in the last 650,000 years. The values measured at Mauna Loa Observatory, Hawaii, increased in 2020 to the unprecedented level of 415 ppm [1]. The unfolding climate catastrophe caused by surplus CO_2_ in the atmosphere is an immediate threat to our collective security and prosperity [2]. Therefore, significant effort is being placed on carbon dioxide capture and storage [2,3,4,5]. However, there is also a growing interest in CO_2_ as a valuable and renewable resource that can be used in a wide range of possible application areas from fuels to bulk and commodity chemicals and even to specialty products with biological activity such as pharmaceuticals [6,7,8,9]. The areas of non-isocyanate and phosgene-free polyurethanes are particularly interesting for polymer chemists [10]. The catalytic chemical fixation of carbon dioxide by cycloaddition to oxiranes represents a versatile green chemistry route to environmentally benign multifunctional cyclic carbonates as intermediates for the formation of non-isocyanate polyurethanes [10,11].

Growth of the number of published reports in the area of non-isocyanate polyurethanes was possible due to the development of methods of synthesis of five-membered alkylene carbonates (1,3-dioxolan-2-ones). There are two main synthetic routes leading to these compounds: the reaction of a respective oxirane with carbon dioxide or 1,2-diol with dialkyl or diphenyl carbonate. Instead of carbonic acid esters, phosgene or its derivatives can also be used. The insertion of gaseous carbon dioxide into the oxirane ring, however is still the most convenient method of obtaining five-membered cyclic carbonates. Recently published review articles concerning addition of CO_2_ to oxiranes are given in [12,13,14].

The most represented catalytic systems used for oxirane carbonation are based on simple transition-metal complexes or, more recently, on complex bimetallic ones [12,15]. These catalysts are required in large amounts and need harsh reaction conditions. Introduction of transition-metal complex binary systems consisting of a metal complex and an ammonium salt or tertiary amine co-catalyst allowed significant lowering of the catalyst amount, although the reaction still required pressures exceeding 1 MPa and temperatures above 100 °C [16,17,18]. Another, considerably more efficient group are difunctional catalysts composed of two elements: a transition-metal catalytic site and an ammonium (or phosphonium) ion covalently bound to each other [19,20]. Several works have also presented catalytic systems consisting of the transition-metal sites bound to various solid supports such as montmorillonite clay, starch, magnetic iron oxide particles and other organic and inorganic materials [12].

Furthermore, five-membered cyclic carbonates have been obtained in presence of phosphorus-containing catalysts, ionic liquids, alkali metal halides in presence of various co-catalysts, metal-organic frameworks (MOF’s) or by electrochemical procedures [12]. 

The industrial scale production of five-membered cyclic carbonates, however, is mainly based on quaternary ammonium salts, as well as anion exchange resins as catalysts. There has been only limited progress in development of new catalysts of this type recently, concerning a couple of quaternary ammonium salts on solid supports such as polystyrene, functionalized celluloses, silica gel, inorganic oxides or even carbon nanotubes [12].

Hyperbranched polyglycerols (HBPGs) are structurally defined, biocompatible macromolecular scaffolds, that have an aliphatic polyether backbone, and possess multiple hydroxyl terminal groups [21,22].The hydroxyl groups in polyglycerol are reactive and susceptible to modification, leading to polymers with carboxyl, amine, and vinyl groups, as well as to polymers with bonded aliphatic and perfluorated chains, sugar moieties, and covalently immobilized bioactive compounds, in particular proteins [23]. HBPGs are easily synthesized by ring-opening polymerization of glycidol [24] or glycerol carbonate [25].

In this work we propose a synthesis and characterization of a new type of catalyst containing hydrophobic, CO_2_-philic siloxane residues [26] and quaternary ammonium salts both anchored to a hyperbranched skeleton of polyglycerol. Due to a good solubility of polysiloxanes in supercritical carbon dioxide [27] and limited solubility in polar media we expected to achieve a catalyst that would show fast CO_2_ addition times to oxiranes and be well separable from the reaction products. The results of the catalytic activity were compared to a hydrophilic analogue of the proposed catalyst.

## 2. Materials and Methods 

### 2.1. Materials

All the reagents were purchased from Sigma-Aldrich (Poznań, Poland) and used as received. Solvents were purchased from POCh (Gliwice, Poland) and used as received except for tetrahydrofuran that was used immediately after distillation over potassium benzophenone ketyl (distillation to the reaction flask over argon). Carbon dioxide was purchased from Multax (Zielonki-Parcela, Poland). Ion exchange resins Amberlite^®^ IRC120 H, hydrogen form and Lewatit^®^ MonoPlus MP 500 Cl were purchased from Sigma-Aldrich (Poznań, Poland) and washed thoroughly with methanol prior to use.

Synthesis of aminated polyglycerol A-HBPG was performed according to a previously described procedure [28] 

### 2.2. Instrumentation

FTIR (Fourier transform infrared) spectra were recorded on a Nicolet iS5 Mid Infrared FT-IR Spectrometer equipped with iD7 ATR optical base (Thermo Scientific®, Waltham, MA, USA). ^1^H, ^13^C, COSY (correlation spectroscopy) and HSQC-DEPT (heteronuclear single quantum correlation-distortionless enhancement by polarization transfer) NMR spectra were recorded on a Varian VXR 400 MHz (Palo Alto, Ca, USA) or Bruker AVANCE 500 MHz spectrometers (Bremen, Germany) using tetramethylsilane as an internal standard and deuterated solvents (CDCl_3_, DMSO-d_6_). Samples of 60–100 mg per 0.5 mL of the solvent were used. The average molecular masses of polymers were determined based on ^1^H NMR spectra. The integral of the CH_3_ group signal coming from the core trimethylolpropane (TMP) molecule was used as a reference. Elemental analysis was performed with Elementar Vario EL III CHNS analyzer (Analysensysteme GmbH, Hanau, Germany). DTA/TG measurements were carried out by using Netzsch Jupiter STA 449C coupled with a Netzsch QMS 403C Aeolos mass spectrometer (Selb, Germany). The heating rate was 10 °C min^−1^ and the final temperature was 600 °C. The measurements were performed in the constant flow of two gases: argon—10 mL min^−1^ (protective gas) and synthetic air (80:20 N_2_:O_2_)—60 mL min^−1^.

### 2.3. Syntheses

#### 2.3.1. ROP Polymerization of Epoxy Phthalimide and Glycidol

Epoxy phthalimide monomer and its copolymers (**1a–1b**) with glycidol were synthesized according to previously reported procedure [28]. The amounts of reagents and reaction yields are given in Table 1.

**1a**^1^H NMR (DMSO–d_6_, 400 MHz): δ (ppm) = 7.99–7.66 (m, H_AR_), 5.33–4.18 (m, OH), 4.05–2.91 (m, CH, CH_2_), 1.32–1.11 (m, 2H, CH_2_–TMP); 0.84–0.63 (m, 3H, CH_3_–TMP); ^13^C NMR (DMSO–d_6_, 400 MHz): δ (ppm) = 168.1 (C_AR_), 134.4 (C_AR_), 131.9 (C_AR_), 123.1 (C_AR_), 72.3–70.1 (CH_2_), 81.0–80.0 (CH), 62.2–68.1 (CH_2_OH), 73.3–72.1 (CH_2_), 69.4–68.1 (CHOH), 78.7–78.0 (CH), 63.7–62.5 (CH_2_OH), 7.7 (CH_3_–TMP); FTIR (ATR): ν (cm^−1^) = 3367, 2873, 1771, 1705, 1607, 1585, 1467, 1429, 1394, 1191, 1035, 918, 724, 714, 530. The 1b spectrum was analogous showing lower intensity of TMP signals.

#### 2.3.2. Alkylation of 1 with Allyl Bromide

30.34 g (225.4 mmol of OH groups) of polymer **1a** was placed in a 1-L three-necked flask equipped with a magnetic stirrer, a reflux condenser, a thermometer and an argon inlet followed by 450 mL of dry DMF. To a light yellow solution 10.88 g (453.2 mmol) of NaH was added in portions under argon atmosphere. The obtained slurry was stirred at room temperature for 1 h until hydrogen gas was no longer released. Next, the slurry was cooled down to 10 °C and 32.6 g (0.27 mol) of allyl bromide was added dropwise over a period of 2 h. The reaction mixture was stirred at room temperature for 2 h. The excess of NaH was decomposed with small amount of ethanol. The product was extracted with methylene chloride. The organic phase containing the polymer was then washed several times with distilled water to get rid of the DMF. The solution was then dried with magnesium sulfate and the solvent removed on a rotary evaporator. Drying under vacuum yielded 28.54 g of dark yellow viscous oil. Amounts of the reagents and yields are given in Table 2.

**2a**^1^H NMR (DMSO-d_6_, 400 MHz): δ (ppm) = 7.68–6.94 (m, HAr), 6.19–5.60 (s, CH=CH_2_), 5.37–4.87 (dd, CH=CH_2_), 4.29–3.79 (d, CH–O), 3.76–2.98 (m, CH, CH_2_), 1.37–1.18 (m, 2H, CH_2_–TMP), 0.87–0.68 (m, 3H, CH_3_–TMP); ^13^C NMR (DMSO–d_6_, 400 MHz): δ (ppm) = 136.4–133.4 (CH_2_CH=CH_2_), 116.8–114.3 (CH_2_CH=CH_2_), 77.2–75.3 (CH–O), 71.9–67.8 (CH_2_–O), 130.3–125.5 (C_AR_), 7.9–6.3 (CH_3_–CH_2_); FTIR (ATR): ν (cm^−1^) = 3081, 2864, 1677, 1618, 1590, 1567, 1458, 1386, 1260, 1085, 994, 919, 843, 778, 747, 714, 655, 559, 417. **2b** spectrum was analogous showing lower intensity of TMP signals.

#### 2.3.3. Hydrosililation of 2 with Heptamethyltrisiloxane

18.2 g of allyl derivative **2a** was placed in a 1-L three-necked flask equipped with a magnetic stirrer, a reflux condenser, a thermometer and an argon inlet followed by 300 mL of dry toluene. The mixture was heated at 45 °C under argon until complete dissolution of the polymer. Then 100 mg of chloroplatinic acid catalyst was added followed by dropwise addition of 31.6 g of 1,1,1,3,5,5,5-heptamethylotrisiloxane. The reaction mixture was stirred at 45 °C under argon for 9 days. The solvent was removed on a rotary evaporator and the polymer dried with a vacuum pump. The crude dark yellow oily product was purified by column chromatography using ethyl acetate/hexane = 1/3 eluent. Amounts of the reagents and yields are given in Table 3.

**3a**^1^H NMR (CDCl_3_, 400 MHz): δ (ppm) = 8.29–6.85 (m, H_AR_), 4.23–2.68 (m, CH, CH_2_), 1.62–1.49 (m, CH_2_CH_2_Si), 0.89–0.76 (m, 3H, CH_3_–TMP), 0.59–0.27 (m, CH_2_CH_2_Si), 0.25–(−0.26) (m, SiCH_3_); ^13^C NMR (CDCl_3_, 400 MHz): δ (ppm) = 24.3–22.9 (CH_2_CH_2_Si), 14.4–13.3 (CH_2_CH_2_Si), 3.0–1.6 (CH_3_SiO), 1.6–1.0 (CH_3_SiCH_2_), 75.0–74.3 (CH_2_O), 74.0–73.2 (CHO), 0.3–(−0.6) (CH_3_–TMP); FTIR (ATR): ν (cm^−1^) = 2957, 1716, 1619, 1591, 1566, 1394, 1250, 1036, 837, 793, 753, 689, 575. **3b** spectrum was analogous showing lower intensity of TMP signals.

#### 2.3.4. Procedure of Hydrazinolysis of Phthalimide Groups

The amine groups of HBPG **3** polymers were deprotected with hydrazine hydrate. In a 1-L two-necked round bottom flask equipped with a magnetic stirrer, argon inlet and a reflux condenser, 46.3 g of HBPG copolymer **3a** followed by 150 mL of dry THF were placed. To the resulting solution 1.16 g (23.2 mmol) of hydrazine hydrate 65% aqueous solution was added and the mixture was stirred at reflux for 20 h. The mixture was cooled down to room temperature. The product was extracted with hexanes. The organic phase was washed several times with brine and dried with magnesium sulfate. The solvent was removed on a rotary evaporator and dried under vacuum yielding 42.0 g of viscous dark yellow oil. The product was stored under argon in a refrigerator. Amounts of the reagents and yields are given in Table 4.

**4a**^1^H NMR (CDCl_3_, 400 MHz): δ (ppm) = 3.92–3.07 (m, CH, CH_2_), 2.85–2.23 (m, NH_2_–CH_2_), 1.69–1.41 (m, CH_2_CH_2_Si), 0.94–0.68 (m, 3H, CH_3_–TMP), 0.52–0.29 (m, CH_2_CH_2_Si), 0.18–(−0.27) (m, Si–CH_3_); ^13^C NMR (CDCl_3_, 400 MHz): δ (ppm) = 75.0–74.0 (CH_2_, CH), 73.7–72.7 (CH_2_, CH), 29.7 (CH_2_NH_2_), 24.3–22.3 (CH_2_CH_2_Si), 14.1–12.6 (CH_2_CH_2_Si), 2.1–1.3 (OSiCH_3_), 1.3–0.5 (CH_2_SiCH_3_), −0.1–(−0.8) (CH_3_–TMP); FTIR (ATR): ν (cm^−1^) = 2957, 1620, 1588, 1449, 1405, 1251, 1036, 837, 793, 780, 753, 687. The **4b** spectrum was analogous showing lower intensity of TMP signals.

#### 2.3.5. Procedure of Quaternization of Amine Groups

23.4 g of polymer **4a** was placed in a 1-L three-necked flask equipped with a thermometer, a magnetic stirrer, a reflux condenser and an argon inlet followed by 450 mL of chloroform. Then 12.1 g of K_2_CO_3_ was added and the mixture was stirred at room temperature under argon for 10 min. Then 4.44 g of methyl iodide was added dropwise and the mixture stirred for additional 24 h. The solid salts were filtrated off. The filtrate was concentrated on a rotary evaporator and dried with a vacuum pump yielding dark yellow viscous oil.

Polymer **6**, was synthesized in methanol. Due to the partial solubility of inorganic salts in this solvent, the purification was performed using ion exchange resins and methanol as an eluent. Amberlite^®^ IRC120 H was used to remove excess potassium carbonate, while Lewatit^®^ MonoPlus MP 500 Cl allowed the change of anions to chloride ones. The procedure caused partial loss of the product on the cation exchange resin. Amounts of the reagents and yields are given in Table 5.

**5a**^1^H NMR (CDCl_3_, 400 MHz): δ (ppm) = 3.93–2.98 (m, CH, CH_2_), 1.64–1.38 (m, CH_2_CH_2_Si), 0.97–0.72 (m, 3H, CH_3_–TMP), 0.51–0.27 (m, CH_2_CH_2_Si), 0.16–(−0.25) (m, Si–CH_3_); ^13^C NMR (CDCl_3_, 400 MHz): δ (ppm) = 74.9–73.8 (CH_2_, CH), 73.5–72.8 (CH_2_, CH), 30.0–29.3 (CH_2_NH_2_), 24.0–22.4 (CH_2_CH_2_Si), 13.9–13.1 (CH_2_CH_2_Si), 2.3–1.4 (OSiCH_3_), 1.2–0.7 (CH_2_SiCH_3_), −0.1–(−0.8) (CH_3_–TMP); FTIR (ATR): ν (cm^−1^) = 2957, 1621, 1588, 1403, 1251, 1043, 840, 794, 756, 688, 581. The **5b** spectrum was analogous showing lower intensity of TMP signals.

**6**^1^H NMR (400 MHz, DMSO–d_6_): δ (ppm) = 4.20 (m, OH), 3.86–2.60 (m, CH_x_O, CH_x_N), 1.24 (m, CH_2_–TMP), 0.79 (m, CH_3_–TMP); ^13^C NMR (100 MHz, DMSO–*d*_6_): δ (ppm) = 73.0, 70.6, 68.6, 63.1, 61.5(CH_x_O); 53.5(CH_3_N), 22.2 (m, CH_2_–TMP), 7.6 (m, CH_3_–TMP).

#### 2.3.6. Anion Exchange Procedure

Since the reaction of quaternization of amine groups was performed in a large excess of potassium carbonate, we assumed that trimethylammonium residues were accompanied with a mixture of iodide and carbonate anions. The anions were exchanged to chloride, bromide or iodide by simple dissolution of the polymer in chloroform and washing with water solution of potassium chloride, bromide or iodide. The resulting solutions were then evaporated and dried with a vacuum pump. In the case of hydrophilic polymer **6** the ion exchange procedure explained above was applied.

For the catalytic activity tests direct products of quaternization (with no ion-exchange procedure) were also used (Table 7, reactions 8–10).

#### 2.3.7. Synthesis of Ethylene Carbonate

Syntheses of ethylene carbonate using poly(ether-siloxane)s **5a-b** and polyglycidol **6** containing trimethylammonium moieties as catalysts were carried out in 50-mL pressure reactors. The exact amounts and molar ratios of reagents and catalysts are given in Table 7. A magnetic element and weighed amount of the polymeric catalyst were placed in the reactor. Then the reactor was closed and the whole thing weighed. The reactor was placed in a dry ice bath and vacuum-nitrogen operation was carried out three times, leaving vacuum in the reactor. Approx. 5 g of ethylene oxide was then introduced to the reactor. Next it was connected to a CO_2_ cylinder. Approx. 5 g of carbon dioxide was introduced. The reactor was then placed in an oil bath pre-heated to 130 °C. The pressure and temperature inside the reactor were controlled during the process. The reaction was carried out for 4 h. Then the reactor was cooled down to room temperature, opened and weighed. The product of the reaction was analyzed using gas chromatography.

## 3. Results and Discussion

### 3.1. Syntheses and Structure Analysis

The purpose of this work was to build a large molecular system based on polyglycidol, containing trimethylammonium moieties and CO_2_-philic siloxane groups and to examine its catalytic activity in the reaction of ethylene oxide with carbon dioxide. For comparison reasons a hydrophilic analogue of this system was also synthesized. The use of hyperbranched polymers was essential in this case due to their unique chemical and physical properties. Their properties are different from those of linear polymers of the same molecular mass. They show lower flexibility, a significant number of chain-end groups, lower viscosity in solutions and higher solubility in common solvents [29].

The proposed synthetic route towards Amm-HBPG’s has a universal character. It allows synthesis of amine or ammonium containing hyperbranched polyether structures with various substituents that can strongly influence the chemical character (hydrophilicity, solubility, etc.) of the whole macromolecule, adapting it to specific requirements. In this case we concentrated on the oligosiloxane substituents, CO_2_-philic residues, that recently showed good solubility in supercritical carbon dioxide [26]. However various other groups like perfluorated hydrocarbons, oxyethylene or other can be introduced using the same procedure.

**Scheme 1:**
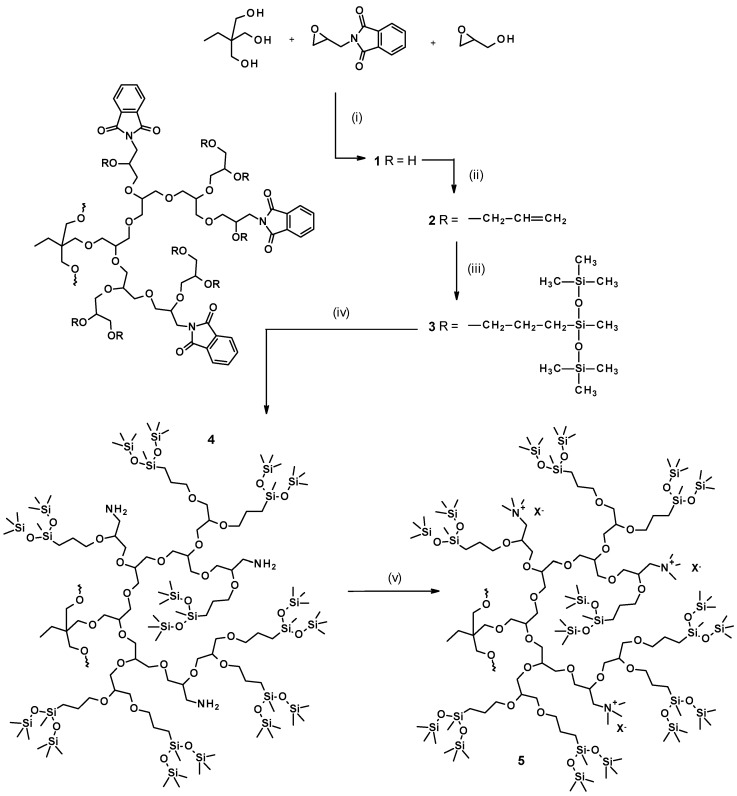
Synthesis of N-(2,3-epoxypropyl)-phthalimide based molecular system **5** containing trimethylammonium residues and multiple oligosiloxane groups: (**i**) TMP, epoxy phthalimide, glycidol, K, THF, 115 °C, 84%–95%; (**ii**) allyl bromide, NaH, DMF, 10 °C, 70%–86%; (**iii**) 1,1,1,3,5,5,5-heptamethylotrisiloxane, chloroplatinic acid, toluene, 59%–77%; (**iv**) 65% hydrazine hydrate, THF, 96%-99%, (**v**) MeI, K_2_CO_3_, chloroform, 91%–93%.

**Scheme 2:**
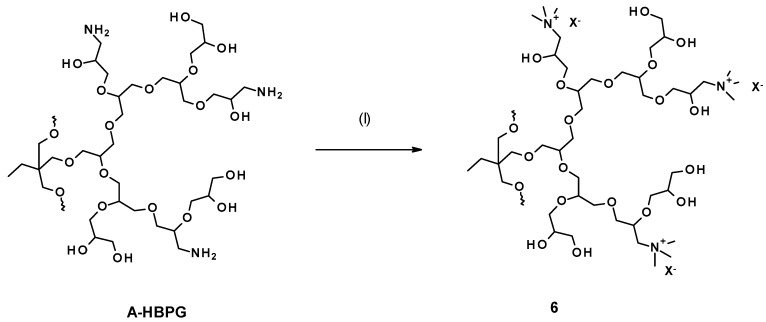
Synthesis of a N-(2,3-epoxypropyl)-phthalimide-based molecular system **6** containing trimethylammonium residues and multiple hydroxyl groups; (**i**) MeI, K_2_CO_3_, chloroform, 91%–93%.

Hyperbranched polyglycerols containing functional trimethylammonium groups and multiple trisiloxane moieties were synthesized in a simple five-step procedure shown in Scheme 1. In the first step a mixture of epoxy monomers was copolymerized in an anionic ring opening polymerization using trimethylolpropane (TMP) as a core molecule and potassium as a deprotonating agent. Application of a core molecule made it possible to control the molar masses and helped to reduce the dispersity of the hyperbranched products [30,31]. Polymers of low and high molar mass were prepared. The first group was characterized by a 10 to 1 molar ratio of epoxy monomers to TMP initiator, the second one by 50 to 1 ratio, respectively. The resulting polymers containing phthalimide and free hydroxyl groups (**1a-b**) were then reacted with allyl bromide in the presence of sodium hydride in dry DMF to yield allyl derivatives (**2**). Unsaturated double bonds of **2** then underwent a hydrosilylation reaction with heptamethyltrisiloxane in the presence of chloroplatinic acid. Next, the amine groups were deprotected via hydrazinolysis of phthalimide residues. The final products **5a-b** were prepared by quaternization of amine groups with methyl iodide. The same reaction was used to obtain hydrophilic analogue **6** from previously synthesized aminated polyglycerol A-HBPG. (Scheme 2)

#### 3.1.1. Monomer

**Figure 1:**
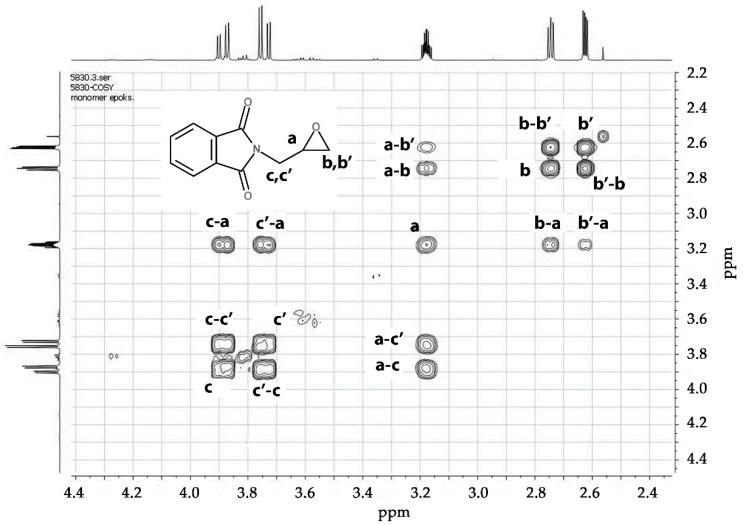
The ^1^H–^1^H COSY (500 MHz, DMSO–d_6_) NMR spectrum of epoxy phthalimide monomer.

**Figure 2:**
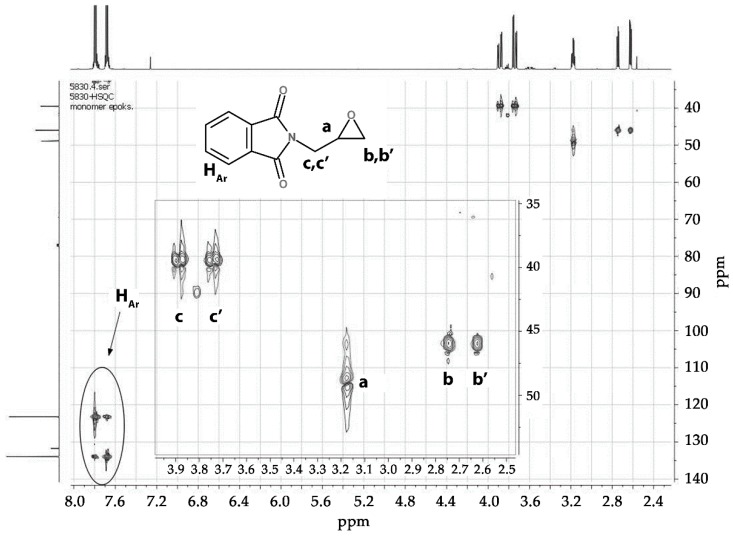
The ^1^H–^13^C HSQC—DEPT (500 MHz, DMSO–d_6_) NMR spectrum of epoxy phthalimide monomer.

The monomer N-(2,3-epoxypropyl)-phthalimide shown in Scheme 1 was obtained in the reaction of potassium phthalimide with an excess of epichlorohydrin and purified by crystallization from methanol [28,32]. Its spectral characterization are given in the supplementary materials (Figure S1–S3) and in ref [28]. Figure 1 and Figure 2 show its COSY (correlation spectroscopy) and HSQC–DEPT (heteronuclear single quantum correlation–distortionless enhancement by polarization transfer) NMR spectra that have not been published before and are crucial to the analyses of polymers containing these structures.

In the structure of the epoxy phthalimide monomer all the aliphatic protons are characterized by different chemical surroundings, therefore its proton NMR spectrum is quite complex and consists of seven groups of signals. Two groups of aromatic protons appear at approx. 8.0–7.6 ppm. The ^1^H–^1^H (COSY) spectrum (Figure 1) clearly shows coupling between aliphatic protons marked as c, c’ and b, b’ within two exo- and endocyclic methylene groups. Coupling between c, c’ and b, b’ protons is not observed due to the too-large distance between them. The signal of the methine proton a connected to the chiral carbon atom shows couplings with both c, c’ and b, b’ protons. On the other hand the ^1^H–^13^C HSQC spectrum (Figure 2) shows the coupling between the ^1^H and ^13^C nuclides. A methylene group belonging to the oxirane ring can be easily distinguished from the one attached to the nitrogen atom.

#### 3.1.2. Copolymerization

The epoxy monomers glycidol and epoxy phthalimide were copolymerized in an anionic ring-opening polymerization using trimethylolpropane (TMP) as a core molecule and potassium as a deprotonating agent. Presence of an ethyl group in the core was important since its separated signals helped in determination of an average molar mass using ^1^H NMR spectroscopy. However, the intensity of the core protons decreased quickly with the growing molar mass of the polymer, and so decreased the accuracy of this method.

**Figure 3:**
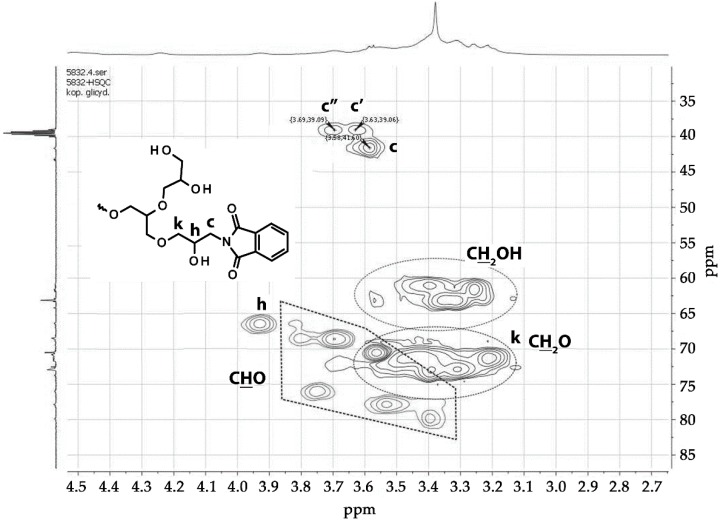
^1^H–^13^C HSQC NMR (DMSO–d_6_, 500 MHz) spectrum of **1a** copolymer.

Hyperbranched polymers **1a-b** were prepared by a modified procedure previously reported by Sunder et al. [24]. The ring-opening copolymerization (ROP) of epoxy phthalimide and glycidol was carried out in bulk, in the presence of TMP and a catalytic amount of potassium. The molar ratio of epoxy monomers to TMP was equal 1 to 10 for 1a polymer and 1 to 50 for 1b polymer. The molar ratio of glycidol to epoxy phthalimide was kept at a constant 3 to 1 level. Earlier studies showed that in reactions mixtures containing more than 25% of epoxy phthalimide, incomplete conversion of this monomer was observed and removing it from the product required a tedious multi-precipitation procedure. The detailed synthetic procedure and characterization of 1a-b copolymers is given in [28]. Additional evidence of the incorporation of epoxy monomer to the structure is the HSQC spectrum shown in Figure 3.

Appearance of three c signals of methylene groups attached to the imide nitrogen atom show the presence of three possible substructures belonging to liner and terminal units. Separate groups of signals of methylene and methine groups attached to hydroxyl or ether oxygen atoms are also visible.

#### 3.1.3. Introduction of Allyl Groups

Polymers **1a-b** were next reacted with allyl bromide in dry DMF using sodium bromide as deprotonating agent. Reaction yields ranged from 70% to 86% and were higher for the higher molar mass **2b** polymer. In case of **2b** the fraction of low molar mass lost during multiple washing procedure was smaller. It was important to have all the hydroxyl groups fully substituted with allyl residues. In the other case, the washing process was very time consuming due to emulsion formation of amphiphilic molecules containing both hydrophilic hydroxyl and hydrophobic allyl groups.

**Figure 4:**
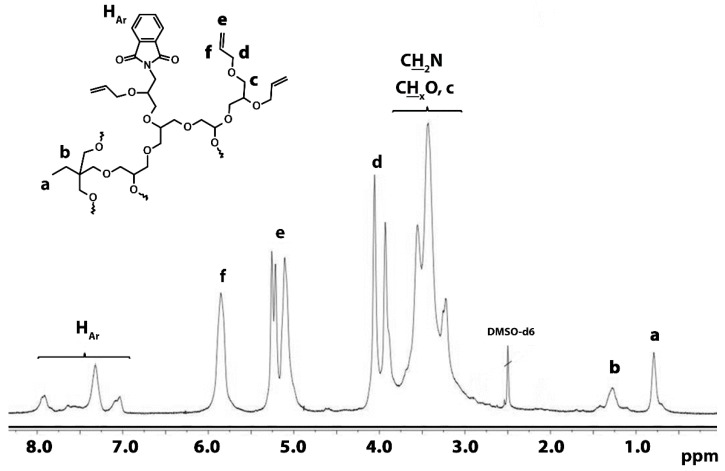
^1^H NMR (DMSO–d_6_, 500 MHz) spectrum of **2a** copolymer.

Figure 4 shows a ^1^H NMR spectrum of **2a**. The spectrum contains four groups of signals. The starting TMP unit signals are present at 0.7 and 1.3 ppm, the hyperbranched polyether structure protons are grouped in the 3.0 to 3.7 ppm area. Next the protons of allyl residues are observed in the 3.7 to 6.0 ppm range and aromatic protons in the 6.9 to 8.0 ppm range. The FTIR spectrum of **2a** (Figure S4) did not show the absorption band at 3300 cm^−1^ confirming full substitution of hydroxyl groups. The ^13^C, COSY (Figure S5) and HSQC (Figure S6) spectra gave further information on the structure of the polymer. The structure and signal assignments are given in supplementary materials. Integration of the signals in the ^1^H NMR spectrum showed that the ratio of units containing aromatic structures to allyl ones to TMP was equal 2 to 10.3 to 1. This corresponds to the macromolecules of average molecular mass equal 1490 g/mol and average nitrogen concentration on the level of 1.88%, which was slightly lower than theoretical one, but higher than the value obtained by elemental analysis. The theoretical molar masses and nitrogen concentrations in most of polymer samples were collected and are compared with experimental ones in Table 6.

Due to the lack of appropriate standards and the solubility issues, the use of size-exclusion chromatography (SEC) measurements for the analysis of hyperbranched polymers is questionable. Based on our experience and literature reports we have applied NMR spectroscopy for the characterization of the polymers investigated in this work. The data collected in Table 6 shows good agreement between the theoretical and NMR-calculated average molecular masses for polymers **1a** and **2a**. However, the amounts of aromatic units were found to be a little lower than expected. A possible explanation of this finding may be that the preparation procedure causes loss of some low-molar mass-oligomers.

#### 3.1.4. Hydrosililation of Allyl Groups with Heptamethyltrisiloxane

The hydrophobic siloxane CO_2_–philic residues were incorporated to the structure of the polymer by reaction with 1,1,1,3,5,5,5-heptamethyltrisiloxane in the presence of a catalytic amount of hexachloroplatinic acid. NMR spectra (within the accuracy of the method) confirmed conversion of all allyl groups into trisiloxane derivatives. Due to the large size of introduced residues the molecular mass of the polymers increased significantly. Therefore the share of the phthalimide units in the whole material decreased to the level at which they could hardly be seen on NMR spectra and their signals could hardly be used for analytical purposes. However, the integration analysis in this case showed the molar ratio of TMP to glycidol-derived units to phthalimide units to be 1/7.2/1.75. This composition was in agreement with elemental analysis showing 0.69% of N in the sample (Table 6). The **3b** sample showed concentration of nitrogen atoms equal to 0.87%.

**Figure 5:**
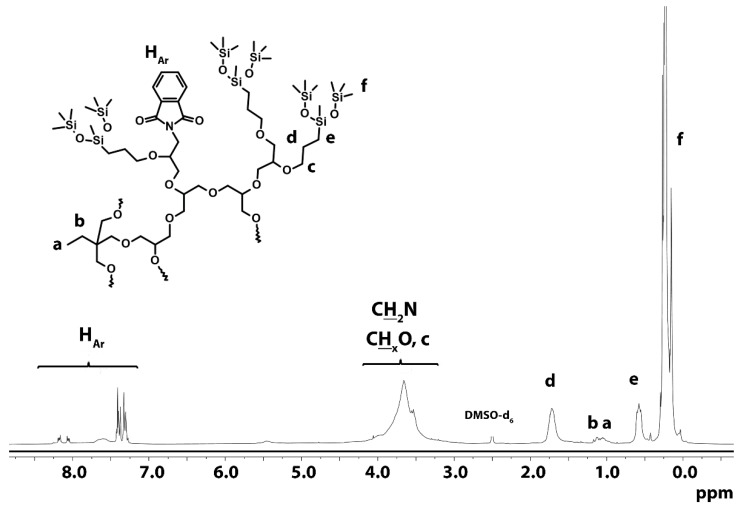
^1^H NMR (DMSO–d_6_, 500 MHz) spectrum of **3b** copolymer.

Figure 5 presents a ^1^H NMR spectrum of polymer **3b** showing no signals of protons situated next to unsaturated bonds. FTIR spectra of **3a** and **3b** (Figure S7, supplmentary materials) were the same and showed mainly absorption bands typical for polysiloxane and polyether structures. COSY and HSQC NMR spectra showed mainly correlations between polyether backbone and siloxane residues, however, the aromatic phthalimide structures were also visible (Figures S8 and S9).

#### 3.1.5. Hydrazinolysis

Aminated polyglycerols (**4a-b**) were obtained in the reaction of copolymers **3a-b** with 65% aqueous hydrazine hydrate performed in ethanol at reflux. In general, the procedure described in the literature was followed [28]. Due to the presence of large number of siloxane residues the polymers **4a-b** showed good solubility in solvents of low polarity. Therefore all the polar by-products and impurities were removed by extraction of the reaction mixture with hexanes and washing the resulting solution with DI water. Removal of the solvent yielded products **4a-b** with no need for further purification.

**Figure 6:**
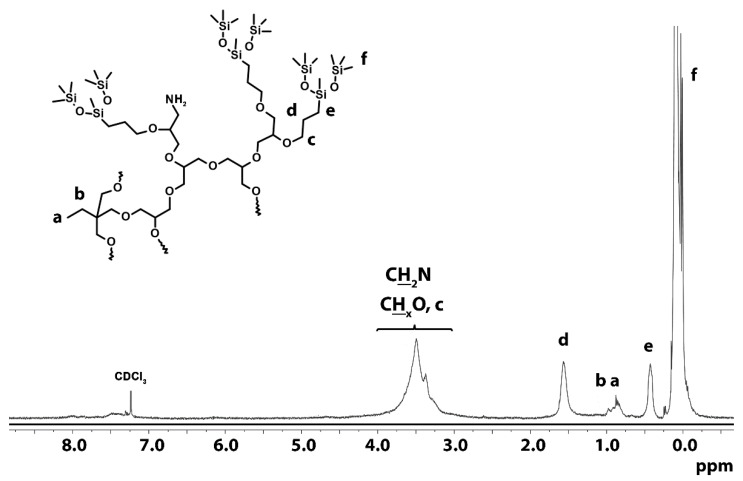
^1^H NMR (CDCl_3_, 400 MHz) spectrum of **4b** copolymer.

The ^1^H NMR spectrum of **4a** (Figure 6) showed the disappearance of signals of aromatic protons after the reaction. The correlation COSY and HSQC confirmed the lack of aromatic signals too. However no distinct signals could be assigned to methylene groups next to amine groups due to their low concentration and their overlapping with polyether backbone signals. The integration of the ^1^H NMR spectrum of **4a** showed the molar ratio of TMP to glycidol-derived units to amine-containing units to be 1/7.3/2, which is equivalent to 0.8% concentration of N atoms. The elemental analysis of samples **4a** and **4b** showed an increase in nitrogen concentration in the polymer in comparison to **3a-b**, respectively up to 0.85% and 1.04% (Table 6). This confirmed the successful deprotection of phthalimide residues.

#### 3.1.6. Quaternization of Amine Groups with Methyl Iodide

Several methods were tested to convert amine groups into trimethylalkylammonium ones. The most successful procedure required the excess of methyl iodide and potassium carbonate as a catalyst and hydrogen chloride scavenger. The reaction was performed in chloroform for derivatives **5a** and **5b** or in methanol for amine-containing polyglycerol (A-HBPG, [28]).

In the case of derivatives **5a** and **5b**, after completion of the reaction, the solid salts were filtered off and the organic solution evaporated to dryness. A sample of such **5b** product was saved and its catalytic activity was checked. Next the polymers were re-dissolved in chloroform and washed with concentrated solutions of potassium halides to yield chloride, bromide and iodide form of the hyperbranched polymer. The NMR and FTIR spectra of **5a** and **5b** polymers with different anions (Cl^−^, Br^−^, I^−^) were very similar to these of **4a** and **4b**. It was necessary to obtain the nitrogen concentration in these samples by elemental analysis. The results ranged from 0.71% to 0.83%. The nitrogen content in these samples decreased in comparison to **4a** and **4b** due to introduction of additional methyl groups to the structure (Table 6).

Hydrophilic polymer **6** was purified using ion-exchange resins in methanol. First a cationite Amberlite^®^ IRC120 H was used to remove excess of potassium carbonate, then the anionite Lewatit^®^ MonoPlus MP 500 Cl was used to unify the type of anions in the sample. The procedure yielded partial loss of the product due to the adsorption on a cation-exchange resin, but was necessary to get rid of all of the inorganic salts. It should be noted that the procedure of multiple precipitation of the polymer from methanol/ethanol mixture used previously was ineffective [28]. 

**Figure 7:**
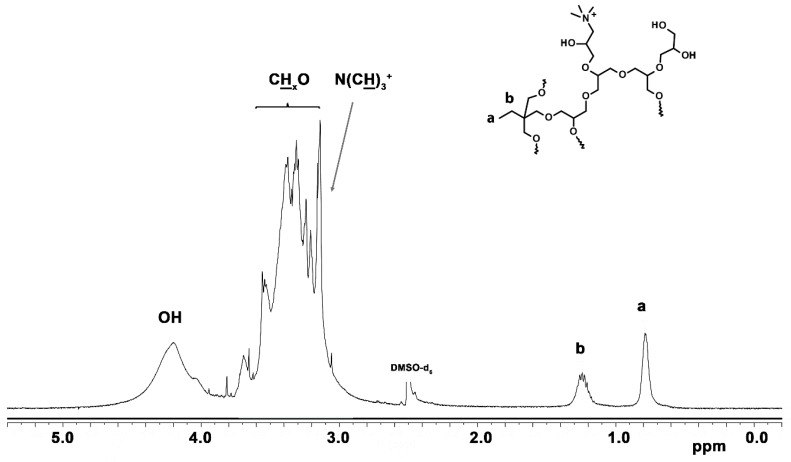
^1^H NMR (DMSO–d_6_, 400 MHz) spectrum of **6** copolymer.

Figure 7 presents a ^1^H NMR spectrum of the polymer **6**. In this case the methyl groups adjacent to nitrogen are clearly visible. Integration of the signals in the spectrum is in good agreement with the molar ratio of TMP to glycidol to trimethylammonium units equal 1 to 7.1 to 1.75 (Table 6), which corresponds to 2.66% of N. The value obtained by elemental analysis was very close on the level of 2.51% of N.

DTA/TG thermal degradation measurement of **6/Cl** showed that the sample was thermally stable up to about 248 °C (5% mass loss). It degraded in a single mass loss step and had the largest thermal stability of all three investigated catalysts. Its mass-loss rate reached a maximum at 380 °C. The samples **5a/Cl** and **5b/I** started actively losing mass at 175 and 208 °C, respectively. They degraded in three steps with the highest mass loss rate at 380 °C as well. These results show that the catalysts are thermally stable under conditions of the reaction of cycloaddition.

### 3.2. Catalytic Activity of Hyperbranched Polymers

**Scheme 3:**
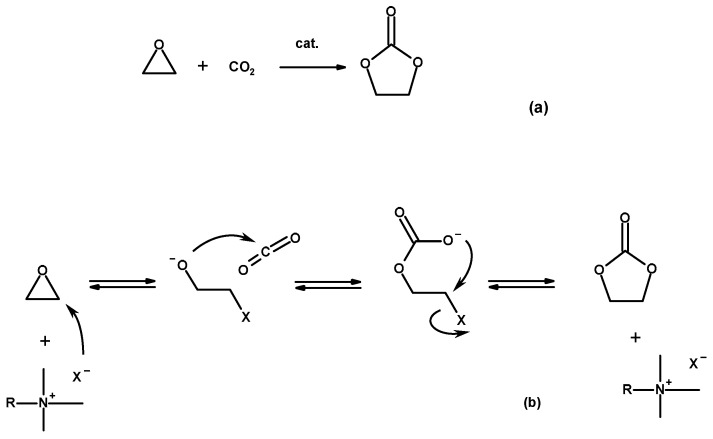
Cycloaddition of carbon dioxide to oxirane (**a**), and a mechanism of the reaction (**b**).

The catalytic activity of polymers **5a-b** and **6** was tested in the rection of ethylene oxide with carbon dioxide. The mechanism of this reaction is given in Scheme 3. Both of the starting materials are in a gaseous phase at the reaction conditions. Before formation of the liquid phase of the product the reaction rate can be very slow. Therefore we proposed to introduce the CO_2_-philic, siloxane-containing polymeric matrix that attracts carbon dioxide and enhances the catalytic activity of the trimethylammonium moieties. The advantage of the synthesized hyperbranched polymeric catalysts was their low viscosity. They were liquid at the reaction conditions and easily dissolved both of the reagents, allowing their faster interaction.

The reactor containing starting materials and the catalyst was placed in an oil bath preheated to 130 °C. The reaction was continued for 4 h. The effective reaction temperatures inside the reactor were in the range of 110–120 °C. Then the reactor was cooled down to room temperature. The amounts of reagents and reaction yields of the carbon dioxide addition to oxirane are given in Table 7. To compare various catalysts the amount of nitrogen, corresponding to the number of catalytically active trimethylammonium species in each sample is given. The molar ratio of trimethylammonium groups was kept low in the range between 0.5 to 18 mmol per mole of ethylene oxide, while the molar ratio of carbon dioxide to ethylene was close to 1. The repeatability of the synthetic procedure was checked on example of 5a/Cl and 6/Cl catalysts. Five experiments (R1–R5, Table 7) performed with the same amount of reagents and reaction conditions gave products with yields that differed by not more than 3% in case of **5a**/Cl. Two experiments for **6**/Cl (R6–R7, Table 7) showed yields that differed by a fraction of 1%. The possibility of catalyst recycling was also checked. Reactions marked C1–C3 were performed with the same catalyst separated from the product and reused. In case of catalyst **5b**/I the reaction yield dropped down from first to second run from 73% to 53% and then remained on the same level after the third run. The catalysts **5a** and **5b,** that had deposited at to the bottom of the reactor, were easily separable from ethylene carbonate. Their high viscosity at the melting point of ethylene carbonate (EC) allowed for easy removal of the liquid product. In contrast to siloxane **5a** and **5b** catalysts the hydrophilic **6** catalyst formed a suspension in the product and could not be easily separated.

**Figure 8:**
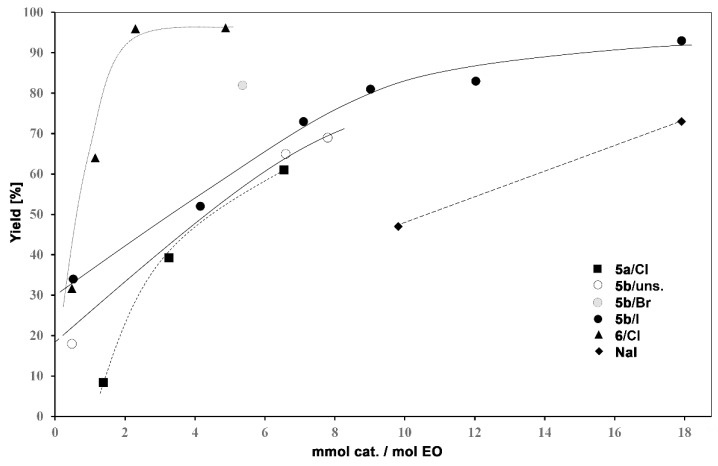
Reaction yields in relation to the type and amount of the catalyst used for carbon dioxide addition (130 °C, 4h).

Figure 8 shows the reaction yields relative to the function of the type and amount of the catalyst used for carbon dioxide addition. All the synthesized catalysts showed higher efficiency than reference NaI catalyst (19, Table 7). Moreover, in all cases, the increase in catalyst amount resulted in increased reaction yield. Surprisingly, the most effective appeared to be the **6**/Cl catalyst bearing free hydroxyl groups. The presence of as low as 2 mmoles of trimethylammonium chloride moieties per 1 mol of ethylene oxide resulted in nearly quantitative reaction yield after 4 h. However, gas chromatography of the product revealed small amounts of ethylene glycol formed as a by-product (from 1.7% for reaction 22% to 2.5% for reaction 25, Table 7). The presence of ethylene glycol (EG) in the product can be attributed to the hydrophilic nature of the catalyst that absorbed water. The vacuum-nitrogen operations performed before introduction of ethylene oxide to the reactor, most probably, was not sufficient to remove all of moisture from the catalyst and contributed to formation of EG.

All the siloxane-modified catalysts **5a-b** showed a moderate increase in the reaction yield with increasing amounts of ammonium species. The highest yield (93%) within the investigated range of catalyst concentrations was achieved for the system **5b**/I (reaction 16, Table 7) containing nearly 18 mmoles of ammonium species per mole of EO. There was only a minor influence of the halide anion observed on the reaction yield, as well as the influence of the average molecular mass of the polymeric catalyst.

The expected higher activity of CO_2_-philic siloxane catalysts **5a-b** over the hydrophilic one **6** was not observed. The possible reason may be insufficiently low solubility of the polymer matrix in CO_2_ under reaction conditions (up to 60 bar). The full homogenization of CO_2_/hyperbranched poly(ether-siloxane) mixture occurs at pressures above 100 bar [26]. Higher activity of the **6** catalyst can be attributed to its lower molar mass and lower viscosity, which allowed fast dissolution of the reactants. The higher mobility of the halide ion in the hydrophilic polymer matrix could also be a reason. However, the presence of hydrophobic **5a-b** matrix prevented formation of ethylene glycol by-product during the process. Figures S10 and S11 in the supplementary materials show the gas chromatography trace of the EC solution in methanol and ^1^H NMR spectrum of the EC product. They show no presence of ethylene glycol signals. In Figure S11 there can be only seen a minute peak at 0 ppm coming from the siloxane methyl groups of the catalyst.

In the case of all polymeric catalysts no distinct pressure drop was observed during the reaction. The temperature and pressure values changed gradually meaning, that the process started already during warming up of the reactor.

Ion-exchange capacities for catalysts **5a**, **5b** and **6** were calculated to be 0.33, 0.51 and 1.79 meq/g respectively. The values for **5a** and **5b** were lower the values for typical ion-exchange resins used in industrial processes (0.8 to 1.8 meq/mL for solvated or 4.3 meq/g for dry resin). The value for **6** catalyst was within this range taking into consideration that the **6** catalyst does not require the use of a solvent. The turnover numbers for obtained catalysts were in the range from 81 for **5a/Cl**, to 104 for **5b/I** and 182 for **6/Cl**. Those values are similar to the values reported for ionic liquids supported on silica (47-140) reported by Han et al. [33].

A sample experiment with propylene oxide (21, Table 1) showed that synthesized catalysts are active in the carbon dioxide cycloaddition reaction of other alkylene oxides. Figure S12 in the supplementary materials shows spectrum of obtained propylene carbonate.

## 4. Conclusions

In this article we reported an easy synthetic route towards hyperbranched polyglycerols containing trimethylammonium and siloxane or hydroxyl end-groups. Polymeric products were characterized using various NMR techniques, FTIR, and elemental analysis. Polymers were shown to be effective in catalysis of addition of CO_2_ to oxirane. Surprisingly, the hydrophilic catalyst showed higher efficiency. However, the siloxane-containing catalysts were easily separable from reaction mixture and showed no formation of ethylene glycol by-product. Obtained polymeric catalysts show high potential in the process of converting carbon dioxide into valuable chemical raw materials and will be further investigated.

## Figures and Tables

**Table 1:** Amounts of reagents used for ring-opening polymerization (ROP) and the reaction yields.

Polymer	TMP/G/P *	TMP	G.*	Pht.*		K	THF	Yield	Yield
	Molar Ratio	g	g	mol %	g	g	mL	g	%
**1a**	1/7.5/2.5	6.02	24.79	22.75	22.72	0.71	125	45.5	84
**1b**	1/37.5/12.5	6.00	124.2	25.0	113.6	0.58	240	232.2	95

* TMP/G/P molar ratio of TMP to glycidol to epoxy phthalimide monomer, G.—glycidol, Pht.—epoxy phthalimide.

**Table 2:** Amounts of reagents used for the reaction of alkylation of **1** and the reaction yields.

Polymer	OH groups	1	NaH	All. Bromide*	DMF	Yield	Yield
	mmol	g	g	g	ml	g	%
**2a**	266.0	30.34	13.0	38.8	450	28.54	70
**2b**	225.4	30.34	10.88	32.65	450	33.65	86

* All. Bromide—Allyl Bromide.

**Table 3:** Amounts of reagents used for the reaction of hydrosililation of **2** and the reaction yields.

Polymer	Allyl groups	2	1,1,1,3,5,5,5-Heptamethyltrisiloxane	Toluene	Yield	Yield
	mmol	g	g	mL	g	%
**3a**	118.1	18.2	31.6	300	26.44	59
**3b**	192.1	33.55	59.1	550	58.44	77

**Table 4:** Amounts of reagents used for the reaction of hydrosililation of **3** and the reaction yields.

Polymer	Phthalimide Groups	3	65% Hydrazine Hydrate w.s.*	THF	Yield	Yield
	mmol	g	g	mL	g	%
**4a**	29	46.3	1.16	150	41.96	99
**4b**	37	48.0	5.1	250	41.22	96

* w.s.—water solution.

**Table 5:** Amounts of reagents used for the reaction of quaternization of **4** and A-HBPG and the reaction yields.

Polymer	Amine Groups	4	K_2_CO_3_	MeI	Chloroform	Yield	Yield
	mmol	g	g	g	mL	g	%
**5a**	16	23.4	12.1	4.4	450	23.3	93
**5b**	9	10.6	6.3	2.5	300	10.5	91
**6**	75	24 (A-HBPG)	52.1	20.8	250 (methanol)	22.5	75

**Table 6:** Characterization of obtained polymers.

Polymer	TMP/G/P * Theoret. Molar Ratio	Theoretical Mol. Mass g/mol	Theoret. % N	Elem. Analysis %N	TMP/G/P * NMR Molar Ratio	NMR Mol. Mass g/mol	NMR %N
**1a**	1/7.5/2.5	1200	2.92	2.67	1/7.4/2.48	1190	2.92
**2a**	1/7.5/2.5	1620	2.16	1.65	1/7.2/2	1490	1.88
**3a**	1/7.5/2.5	3950	0.88	0.69	1/7.2/1.75	3680	0.67
**4a**	1/7.5/2.5	3630	0.96	0.85	1/7.3/2.0	3500	0.80
**5a**	1/7.5/2.5	3890	0.92	0.46 (Cl^−^)	1/7.3/2.0	3660	0.76
**1b**	1/37.5/12.5	5450	3.21	-	-**	-**	-**
**2b**	1/37.5/12.5	7070	2.47	-	-**	-**	-**
**3b**	1/37.5/12.5	16,080	1.09	0.87	-**	-**	-**
**4b**	1/37.5/12.5	14,460	1.11	1.04	-**	-**	-**
**5b**	1/37.5/12.5	15,750	1.06	0.71 (I^−^)	-**		
**5b**	1/37.5/12.5		1.09	0.83 (Br^−^)	-**	-**	-**
**5b**	1/37.5/12.5		-	0.74 (uns.)	-**		
**6**	1/7.1/2.7	1070	3.53	2.51 (Cl^−)^	1/7.1/2.7	924	2.65

* TMP/G/P molar ratio of TMP to glycidol to epoxy phthalimide derived units; **TMP signal of very low intensity.

**Table 7:** The amounts of reagents and reaction yields for carbon dioxide addition to oxirane.

	Catalyst				Ethylene Oxide		Carbon Dioxide		CO_2_/EO	N/EO	Yield
	Cat./anion	[g]	[%_wt._ N]	[mmol N]	[g]	[mmol]	[g]	[mmol]	[mol/mol]	[mmol/mol]	[%]
1	5a/Cl	0.63	0.46	0.21	6.0	150	7.4	168.2	1.1	1.4	8
2	5a/Cl	1.26	0.46	0.41	5.1	127.5	5.8	131.8	1.0	3.2	39
3	5a/Cl^R1^	2.54	0.46	0.83	5.1	127.5	5.5	125.0	1.0	6.5	61
4	5a/Cl^R2^	2.54	0.46	0.83	5.8	145	5.9	134.1	0.9	5.8	59
5	5a/Cl^R3^	2.54	0.46	0.83	5.0	125	5.7	129.5	1.0	6.7	57
6	5a/Cl^R4^	2.53	0.46	0.83	4.7	117.5	5.7	129.5	1.1	7.1	57
7	5a/Cl^R5^	2.54	0.46	0.83	4.9	122.5	5.8	131.8	1.1	6.8	57
8	5b/uns.	0.13	0.74	0.07	5.5	137.5	5.8	131.8	1.0	0.5	18
9	5b/uns.	1.34	0.74	0.71	4.3	107.5	4.5	102.3	1.0	6.6	65
10	5b/uns.	1.58	0.74	0.84	4.3	107.5	5.9	134.1	1.2	7.8	69
11	5b/I	0.13	0.71	0.06	4.9	122.5	4.8	109.1	0.9	0.5	34
12	5b/I	1.00	0.71	0.51	4.9	122.5	5.2	118.2	1.0	4.2	52
13	5b/I^C1^	1.65	0.71	0.83	4.7	117.5	5.7	129.5	1.1	7.1	73
14	5b/I	2.00	0.71	1.01	4.5	112.5	4.6	104.5	0.9	9.0	81
15	5b/I	2.55	0.71	1.29	4.3	107.5	4.7	106.8	1.0	12.0	83
16	5b/I	3.00	0.71	1.52	3.4	85	4.8	109.1	1.3	17.9	93
17	5b/I^C2^	1.59	0.71	0.81	4.8	120	6.6	150.0	1.3	6.7	53
18	5b/I^C3^	1.52	0.71	0.77	4.1	102.5	5.9	134.1	1.3	7.5	52
19	NaI	0.13	-	0.83^1^	3.4	85	4.1	93.2	1.1	9.8	47
20	5b/Br	1.40	0.83	0.83	6.2	155	6.8	154.5	1.0	5.4	82
21	5b/I^P^	1.00	0.71	0.51	6.4	160	5.2	118.2	0.7	3.2	50
22	6/Cl	0.04	2.51	0.07	6	150	7.3	165.9	1.1	0.5	32
23	6/Cl	0.08	2.51	0.14	5	125	5.6	127.3	1.0	1.1	64
24	6/Cl	0.16	2.51	0.29	5	125	6.2	140.9	1.1	2.3	96
25	6/Cl^R6^	0.34	2.51	0.61	5.2	130	5.7	129.5	1.0	4.7	96
26	6/Cl^R7^	0.34	2.51	0.61	5	125	6.2	140.9	1.1	4.9	95

EO—ethylene oxide,**^1^** the amount of mmoles of the NaI; R1–R7—repeatability tests; C1–C3 reactions performed with recycled catalyst (number of cycles); P—reaction performed with propylene oxide; uns.—the product of quaternization of **4b** before ion exchange procedure.

## Supplementary Materials

The following are available online at https://www.mdpi.com/2073-4360/12/4/856/s1: Figure S1: ^1^H NMR (400 MHz, CDCl_3_) spectrum of epoxy phthalimide monomer. Figure S2: ^13^C NMR spectrum of epoxy phthalimide monomer. Figure S3: FTIR-ATR spectrum of epoxy phthalimide monomer. Figure S4: FTIR-ATR spectrum of allyl derivative **2a**. Figure S5: ^1^H–^1^H COSY NMR (DMSO–d_6_, 500 MHz) spectrum of **2a** copolymer. Figure S6: ^1^H–^13^C HSQC NMR (DMSO–d_6_, 500 MHz) spectrum of **2a** copolymer. Figure S7: FTIR-ATR spectrum of siloxane derivative **3a**. Figure S8: ^1^H–^1^H COSY NMR (DMSO–d_6_, 500 MHz) spectrum of 3a copolymer. Figure S9: ^1^H–^13^C HSQC NMR (DMSO–d_6_, 500 MHz) spectrum of **3a** copolymer. Figure S10: The GC chromatogram of the methanol solution of the product of reaction 14 (Table 1)—ethylene carbonate. Figure S11: ^1^H NMR (400 MHz, CDCl_3_—high concentration, solvent signal not visible) spectrum of the product of reaction 14 (Table 1)—ethylene carbonate. Figure S12: ^1^H NMR (400 MHz, CDCl_3_) spectrum of the product of reaction 21 (Table 1)—propylene carbonate.
