# Supplementary material for: Hyperbranched Poly(ether-siloxane)s Containing Ammonium Groups: Synthesis, Characterization and Catalytic Activity
Source: Polymers (Basel). 2020 Apr 7;12(4):856. doi: 10.3390/polym12040856 (PMC7240551; doi:10.3390/polym12040856)
Supplement: Supplementary file 1 [file polymers-12-00856-s001.pdf]

Hyperbranched Poly(ether-siloxane)s Containing Ammonium Groups – Synthesis, Characterization and Catalytic Activity

Paweł G. Parzuchowski [1]*, Aleksandra Świderska [1], Marlena Roguszewska [1], Karolina Rolińska[1,2] Dominik Wołosz [1] and Mariusz Mamiński [3]

[1] Warsaw University of Technology, Faculty of Chemistry, Noakowskiego 3, 00-664 Warsaw, Poland; pparzuch@ch.pw.edu.pl
[2] University of Warsaw, Faculty of Chemistry, ul. Pasteura 1, 02-093 Warsaw, Poland;
[3] Warsaw University of Life Sciences – SGGW, Faculty of Wood Technology, Nowoursynowska 159, 02-787 Warsaw, Poland
* Correspondence: pparzuch@ch.pw.edu.pl; Tel.: +48-22-234-7317

Supplementary materials

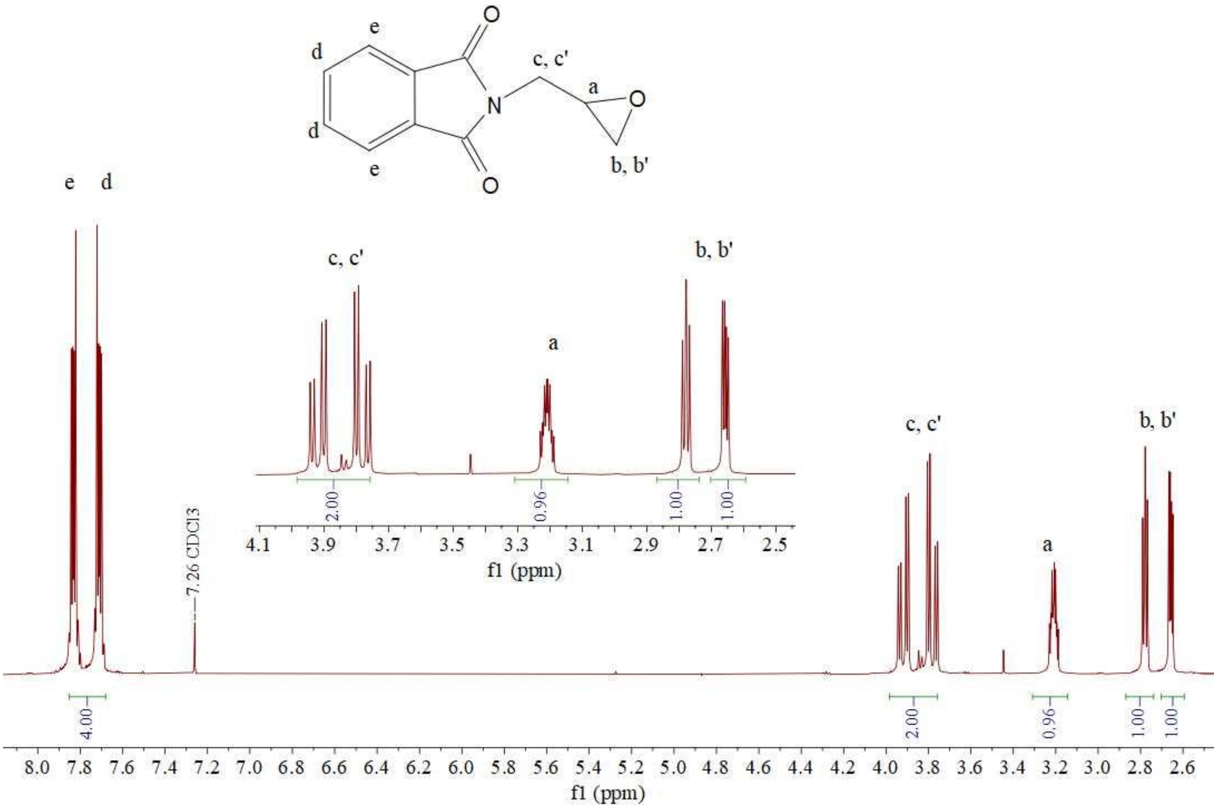

Figure S1. ^{1}H NMR (400 MHz, $CDCl_3$) spectrum of epoxy phthalimide monomer

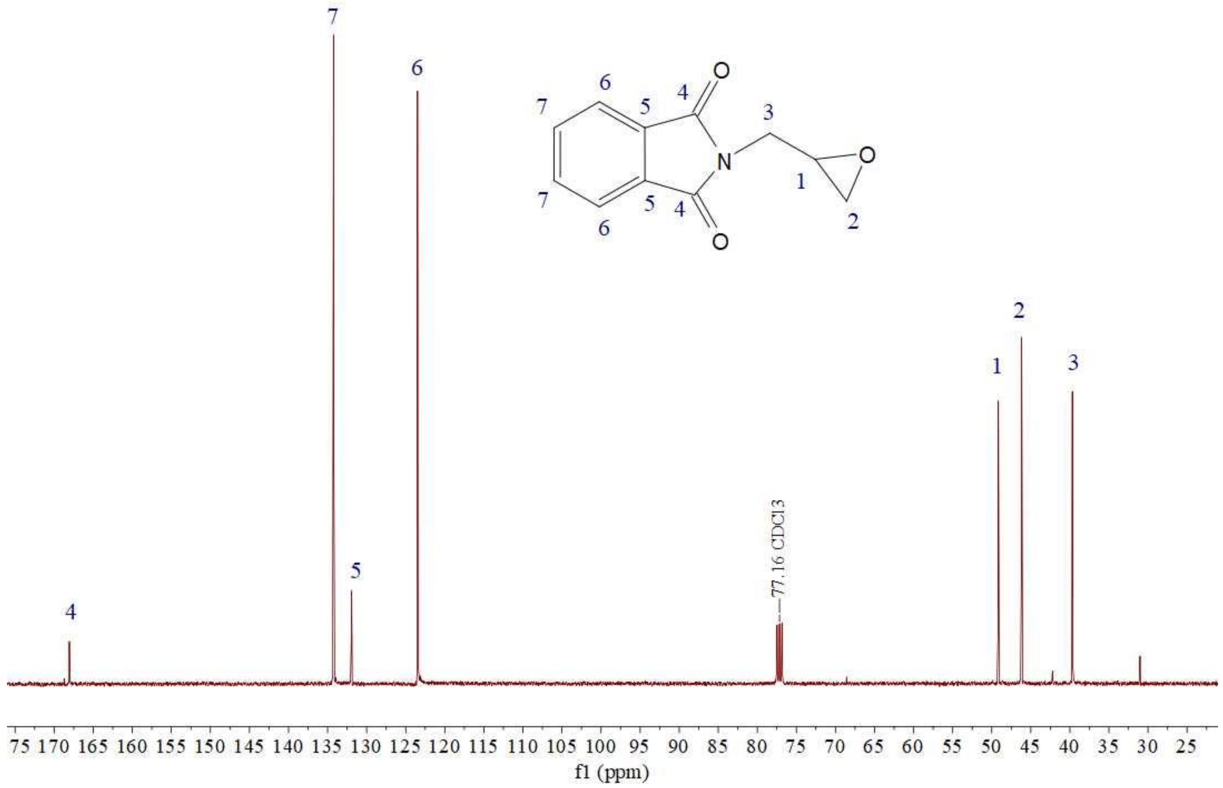

Figure S2. ^{13}C NMR spectrum of epoxy phthalimide monomer

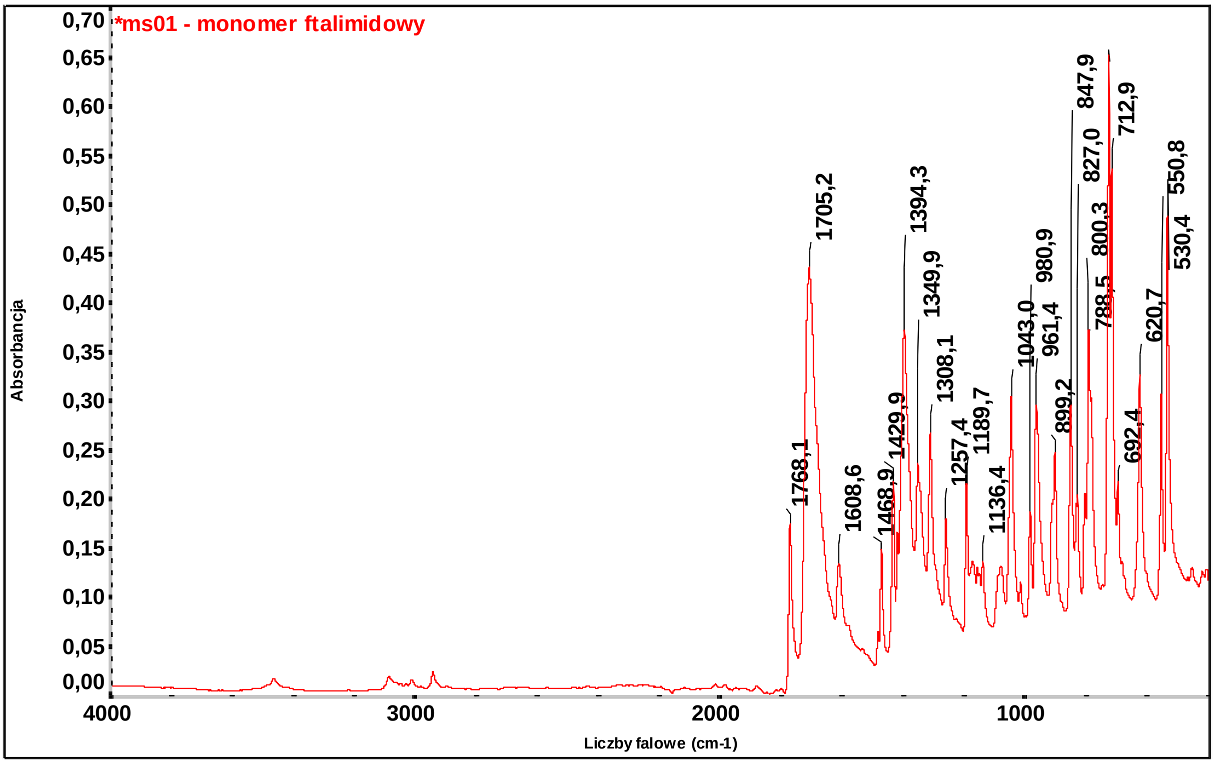

Figure S3. FTIR - ATR spectrum of epoxy phthalimide monomer

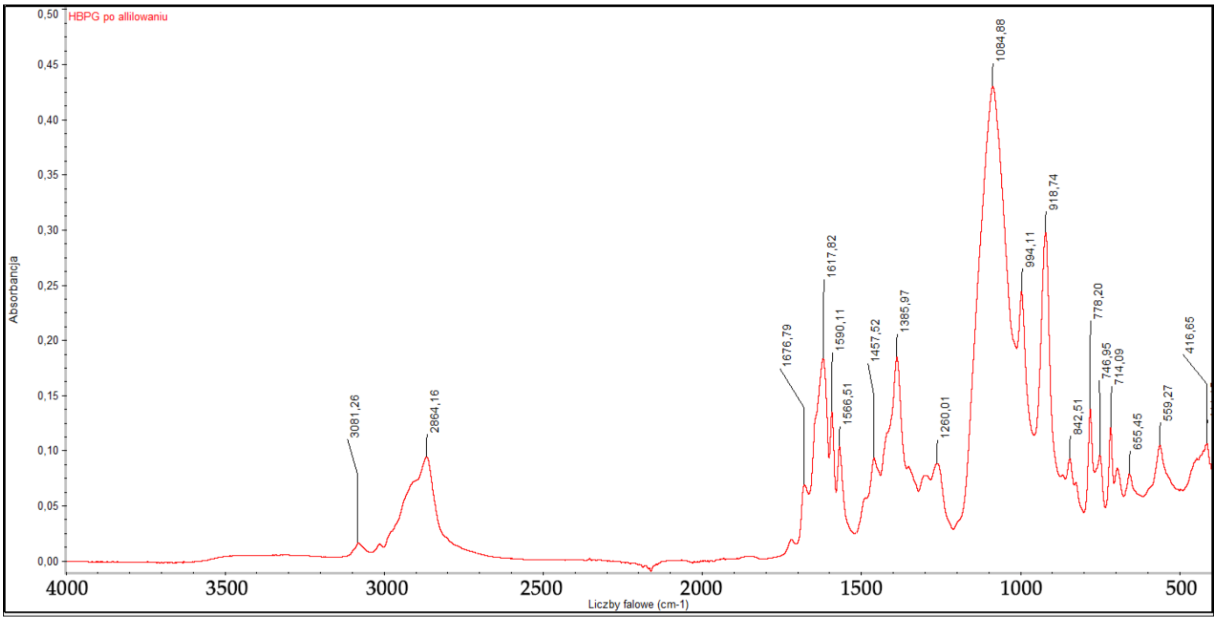

Figure S4. FTIR - ATR spectrum of allyl derivative **2a**

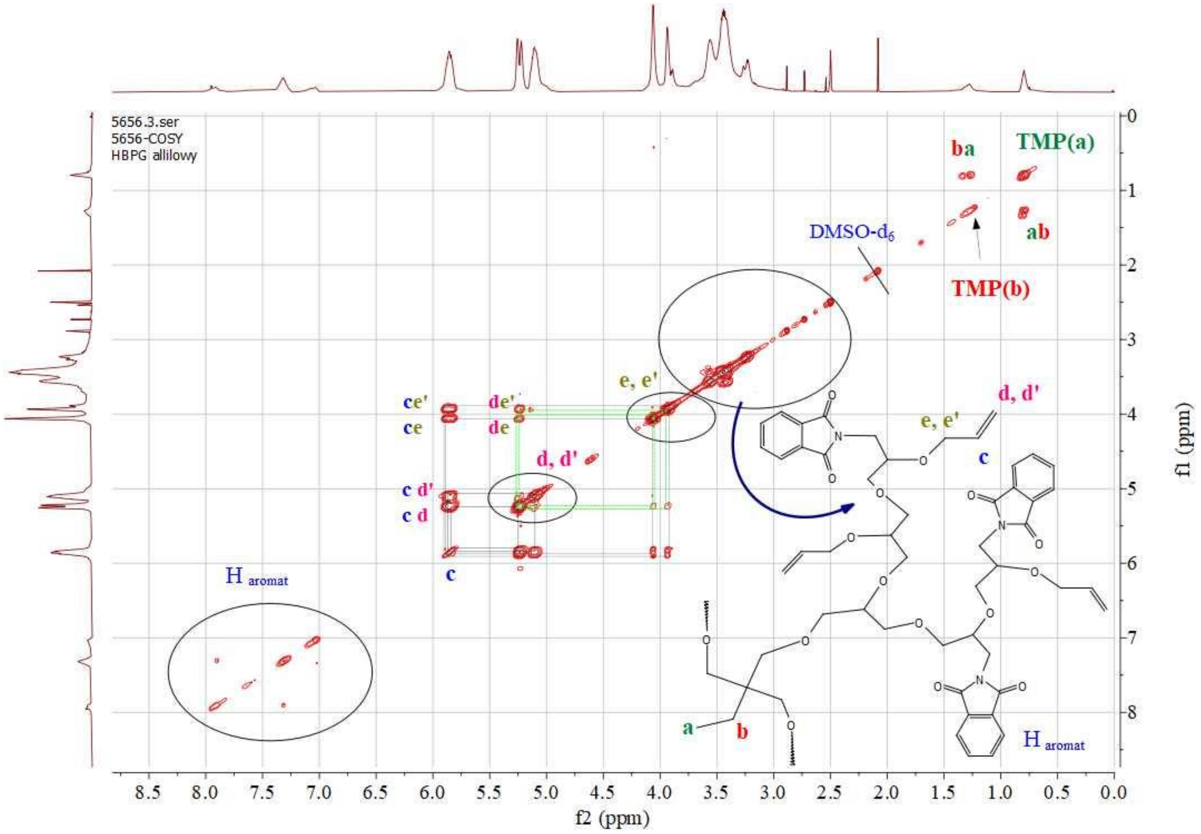

Figure S5. ^{1}H-^{1}H COSY NMR (DMSO-d_6, 500 MHz) spectrum of **2a**copolymer

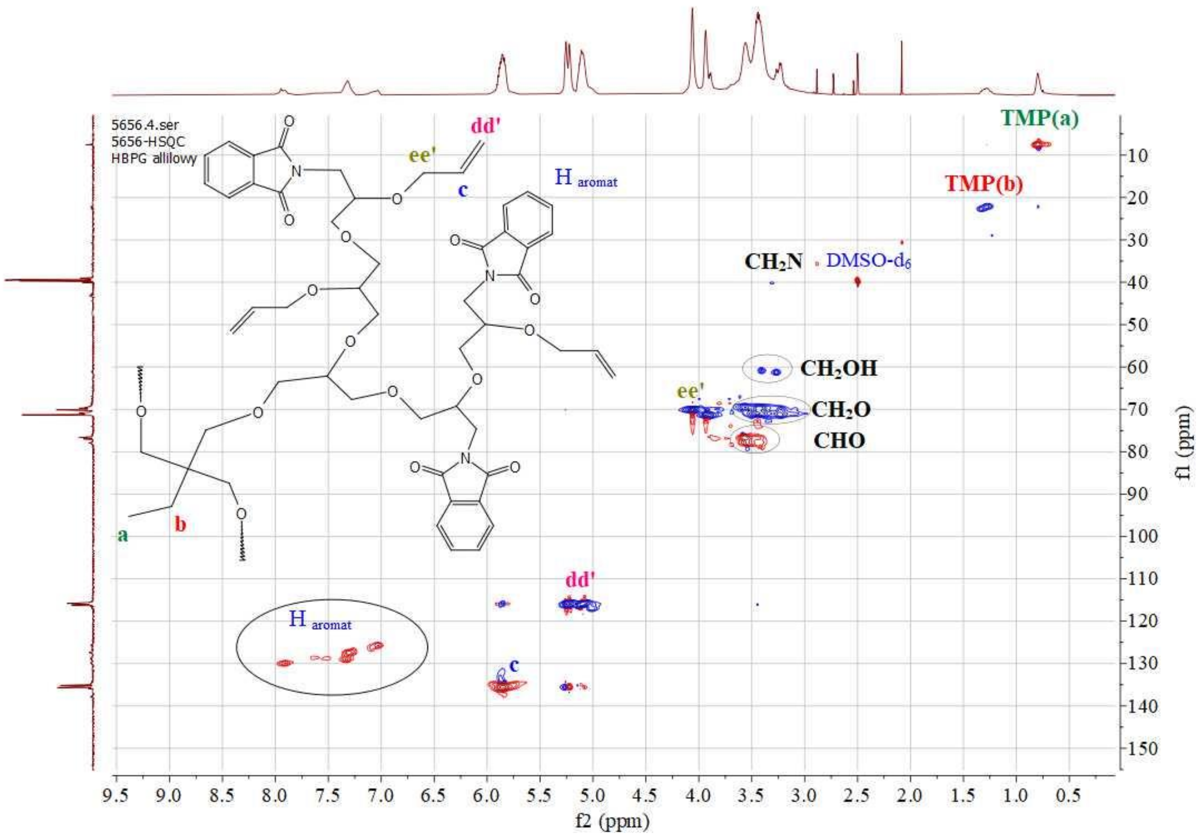

Figure S6. ^{1}H-^{13}C HSQC NMR (DMSO-d_6, 500 MHz) spectrum of **2a**copolymer

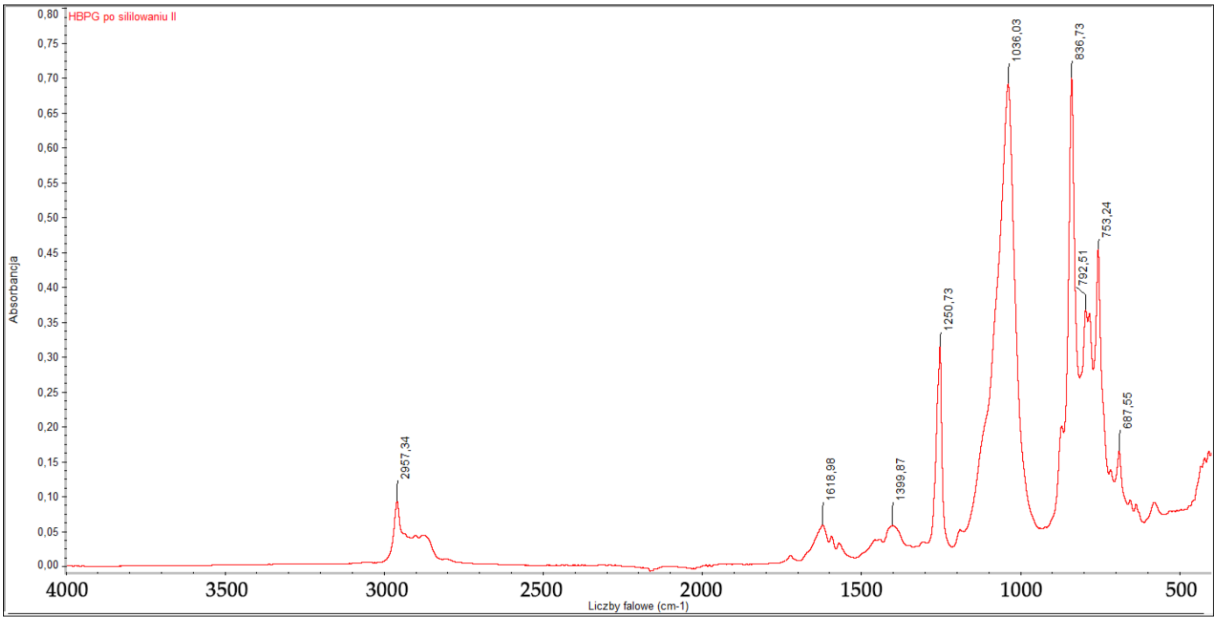

Figure S7. FTIR - ATR spectrum of siloxane derivative **3a**

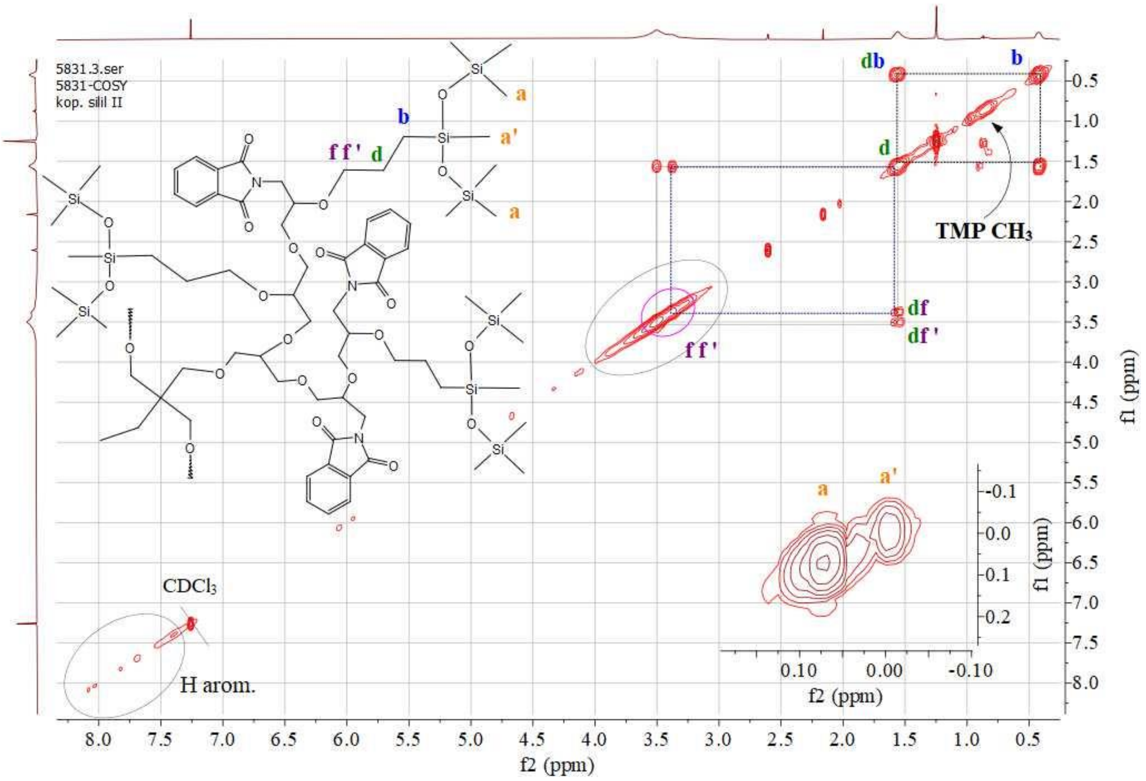

Figure S8. ^{1}H-^{1}H COSY NMR ($CDCl_3$, 500 MHz) spectrum of **3a**copolymer

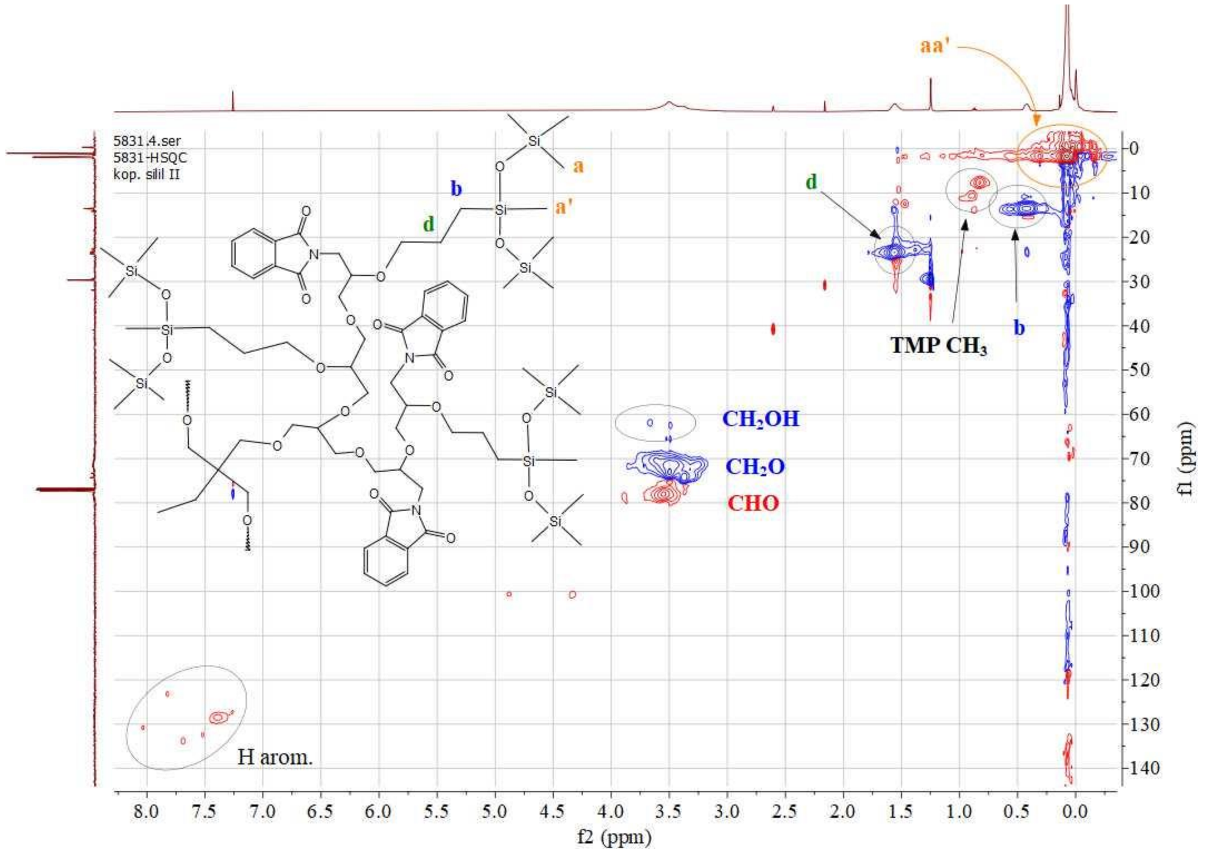

Figure S9. ^{1}H-^{13}C HSQC NMR ($CDCl_3$, 500 MHz) spectrum of **3a**copolymer

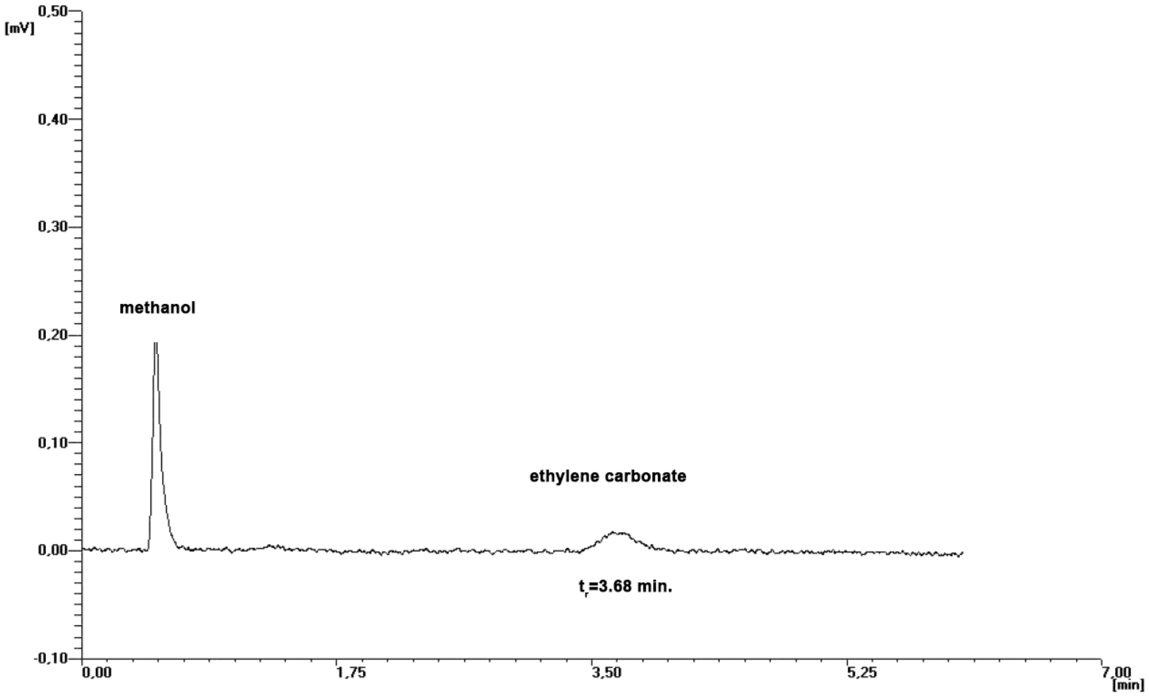

Figure S10. The GC chromatogram of the methanol solution of the product of reaction 14 (Tab.1)– ethylene carbonate

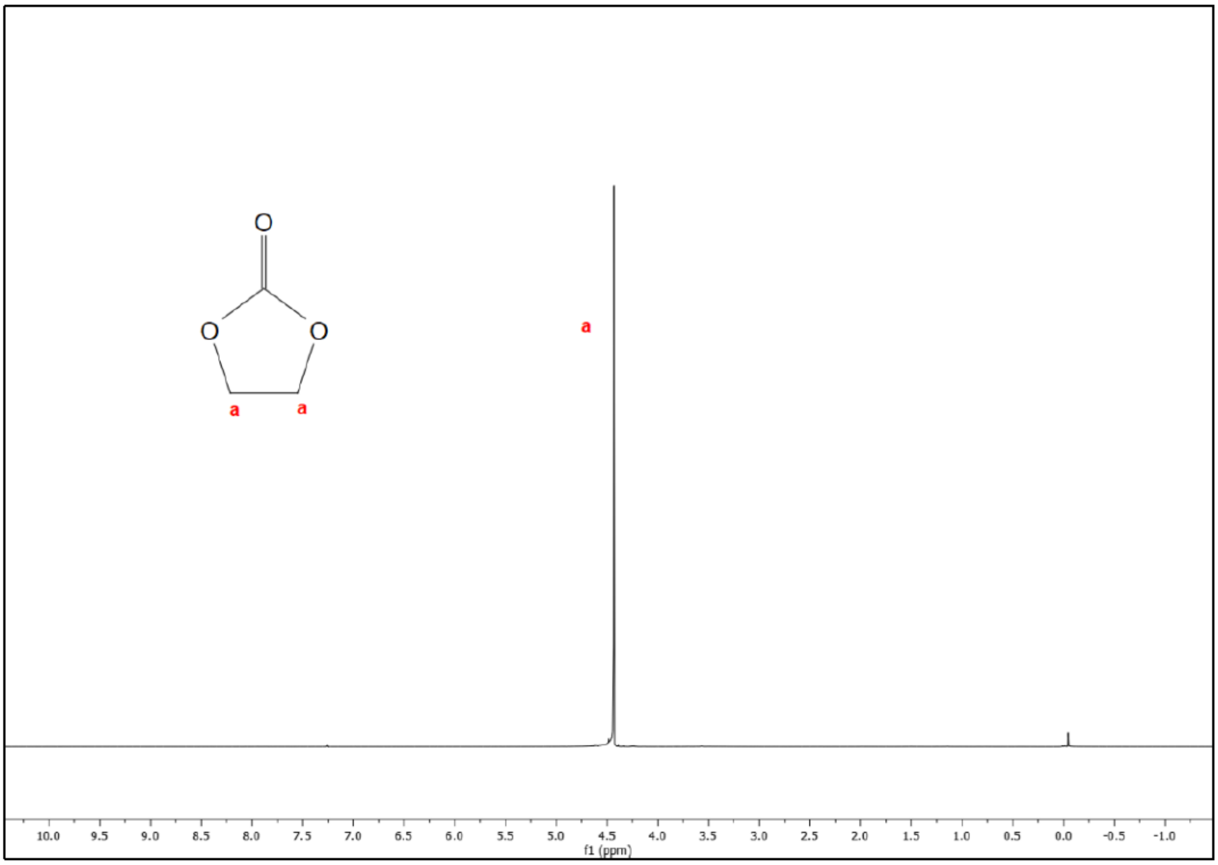

Figure S11. ^1H NMR (400 MHz, $CDCl_3$- high concentration, solvent signal not visible) spectrum of the product of reaction 14 (Tab.1) – ethylene carbonate

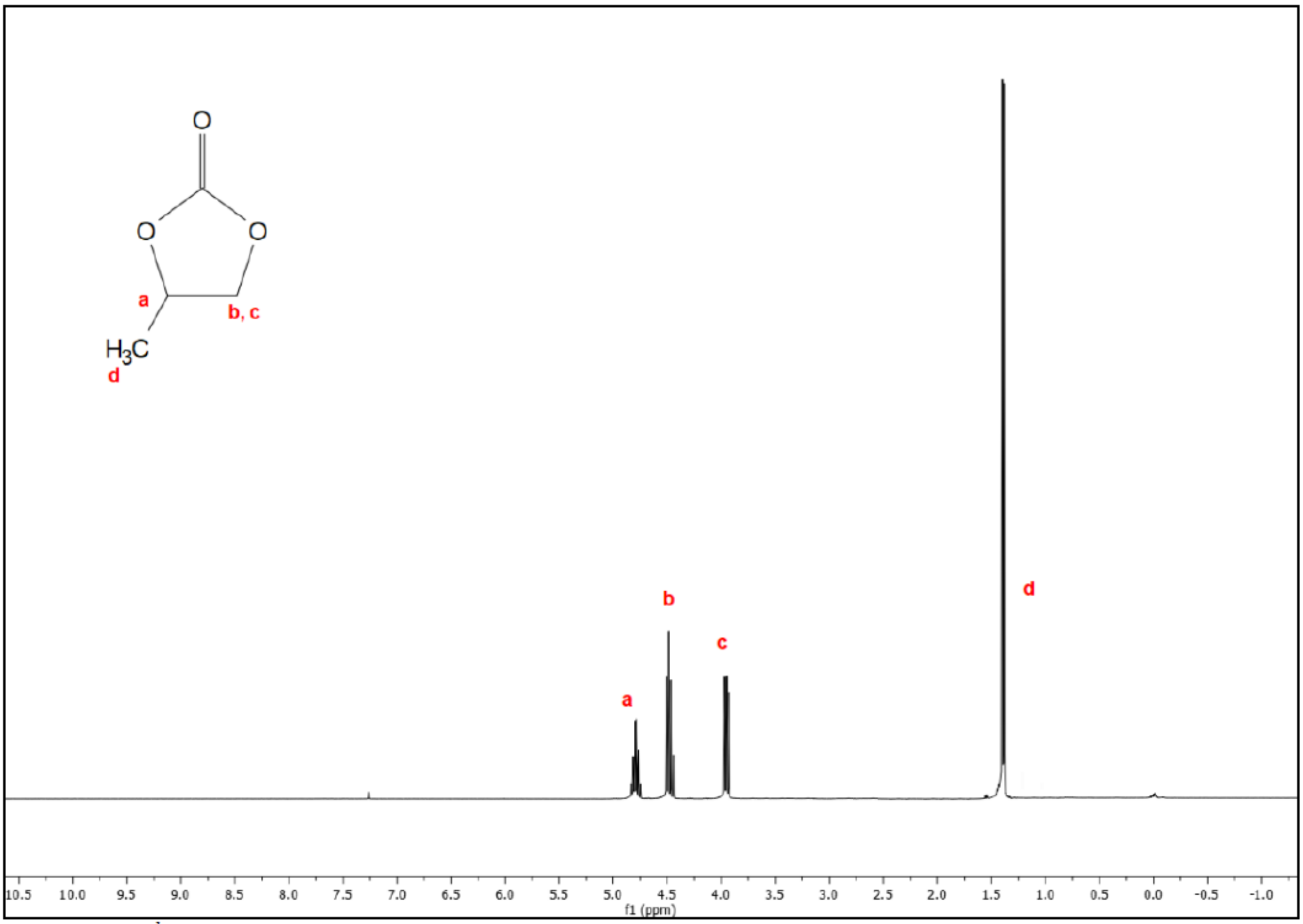

Figure S12. ^{1}H NMR (400 MHz, $CDCl_3$) spectrum of the product of reaction 21 (Tab.1) – propylene carbonate
